# Tissue Dielectric Constant of the Lower Leg as an Index of Skin Water: Temporal Variations

**DOI:** 10.7759/cureus.26506

**Published:** 2022-07-01

**Authors:** Harvey N Mayrovitz

**Affiliations:** 1 Medical Education, Nova Southeastern University Dr. Kiran C. Patel College of Allopathic Medicine, Davie, USA

**Keywords:** edema measurement, lymphedema detection, lymphedema measurement, tdc, tissue dielectric constant, leg lymphedema measurement, lower extremity lymphedema assessment

## Abstract

Tissue dielectric constant (TDC) measurements are a convenient, reliable, and accurate way to noninvasively access local tissue water content and its change with time or treatment. The method has been widely used for upper extremity lymphedema assessments but less so for lower extremities. For lower extremity assessments, it would be useful to have an estimate of the expected inter-leg TDC differentials of normal non-affected legs to help interpret measured inter-leg differentials when such measurements are done in patients with lower extremity edema or lymphedema. The goals of this study were to determine normal inter-leg TDC differences, quantify TDC temporal changes during 60 minutes of supine lying in a group of 10 middle-aged women (42-57 years), and evaluate the change in TDC values as measured throughout the day from 0800 to 2000 hours in a group of 10 younger women (23-28 years). The overall findings indicate that TDC measurements, as an assessment parameter of lower extremity localized skin-to-fat tissue water, are only minimally dependent on potential confounding factors such as 60-minute supine lying or the time of day at which the measurements are made. If the small sample size of the present study is reflective of the larger population, an inter-leg TDC ratio exceeding a value of 1.2 is suggested as a threshold for judging unilateral lower extremity edema or lymphedema. This possibility needs assessment in women with these conditions.

## Introduction

Tissue dielectric constant (TDC) measurements are a convenient, reliable, and accurate way to access local tissue water content and its change since its value strongly depends on skin-to-fat water content [1-4]. The method requires the brief touching of the skin with a probe and has been widely used for evaluating women who are at risk for, or who have already developed, breast cancer treatment-related lymphedema [5-15]. The method has been less used for assessing lower extremity status in healthy women [16,17] or in women who have developed lower extremity edema or lymphedema [18-22]. For lower extremity assessments, it would be useful to have an estimate of the expected inter-leg TDC differentials of normal non-affected legs to help interpret measured inter-leg differentials when such measurements are done in patients with lower extremity lymphedema. Such assessments are often done before starting treatment and then again after treatment, which might be an hour later during which time the patient has been lying supine or near-supine. Since the purpose of pretreatment and posttreatment measurements is to assess treatment outcome, it is important to know how much temporal change might be expected in otherwise unaffected legs because of such lying. Thus, the first goal (goal 1) of the present study was to evaluate inter-leg TDC differences and their temporal changes after one hour of supine lying. Another aspect of temporal variations that is of interest relates to possible variations in lower extremity TDC values dependent on the time of day in which measurements are made. Although there is some information on such effects as measured on upper extremities [23], there is essentially nothing reported concerning the lower extremities. Thus, the second goal (goal 2) of the present study was to provide information bearing on this issue utilizing repeated leg TDC data obtained throughout a day.

## Materials and methods

Subjects

Two different groups of 10 women participated after having each study explained and signing a Nova Southeastern University Institutional Review Board-approved informed consent (#12180901F). To achieve goal 1, women were recruited from faculty and staff according to the following entry criteria: age 18 or greater; no prior history of lower extremity trauma, surgery, edema, or lymphedema; and willing and able to lie supine for 60 minutes. The group of 10 participants had an age range of 42-57 years and a mean ± standard deviation (SD) of 48.4 ± 5.8 years. To achieve goal 2, 10 women medical students were recruited for participation according to the following entry criteria: age 18 or greater and having no prior history of lower extremity trauma, surgery, edema, or lymphedema. These participants were trained in the TDC measurement procedure by the principal investigator. Their age range was 23-28 years (26.2 ± 1.4 years), and they had a normal body mass index (BMI) of 21.7 ± 2.1 kg/m^2^.

TDC measurement

The TDC measuring device (MoistureMeterD compact, Delfin Technologies, Kuopio, Finland) determines the dielectric constant (relative permittivity), which is a dimensionless number equal to the ratio of tissue permittivity to vacuum permittivity. Because TDC values mainly depend on tissue water, they provide quantitative indices of skin water content. The measurement is sensitive to both free and bound water [24]. Inclusion of the bound water contribution is useful since up to 80%-90% of young adult skin water content is bound [25]. The device functions by generating and transmitting a very low power 300 MHz signal into the skin via an open-ended coaxial transmission line [4]. Part of the signal is reﬂected back to permit the calculation of the complex reﬂection coefﬁcient [26] from which the dielectric constant is determined [1]. Reﬂections depend on the complex permittivity of the tissue, which in turn depends on the signal frequency and the dielectric constant (the real part of the complex permittivity), and the conductivity of the tissue with which the probe is in contact. At 300 MHz, conductivity contributes little to the overall permittivity value, and TDC is mainly determined by free and bound water molecules. More details including prior uses for skin assessments, validation, and repeatability data are described in the literature [3,27,28]. Each probe is calibrated against various ethanol-water mixture concentrations each of known dielectric constant values [29]. In this study, measurements were done using a probe that measures a depth of 2.5 mm.

Procedures for goal 1

**Figure 1:**
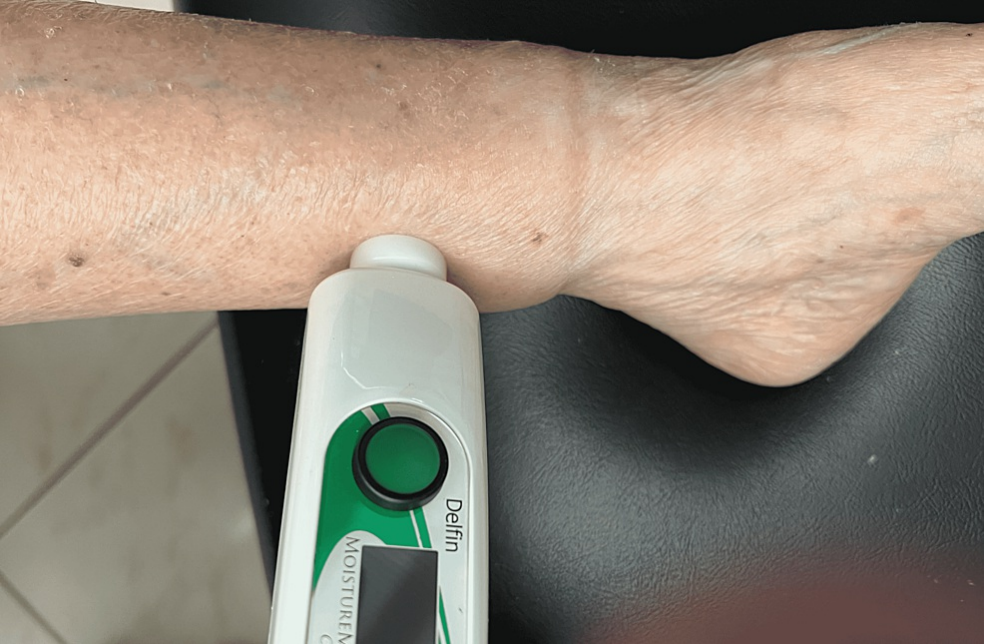
TDC Measurement Illustrated When the probe is placed in contact with the skin, the measurement process is activated and completed within about 4-5 seconds. Measurements at the target site (medial, 8 cm proximal to the medial malleolus) are done in triplicate, and the average value was used to characterize the site TDC value. Measurements are made on both legs at corresponding anatomical sites.

A site on the medial aspect of both legs located 8 cm proximal to the medial malleolus was marked with a skin marker. These sites were the target TDC measurements. After a supine acclimation interval of 10 minutes, TDC was measured in triplicate on both legs with a measurement probe as illustrated in Figure 1. The average of the triplicate measurements was used to characterize the TDC value. The subject remained supine for 60 minutes, after which TDC measurements were repeated. All measurements were made between 1100 and 1300 hours.

Procedures for goal 2

Self-measurements were done by each participant on a day-night interval of 12 hours at two-hour intervals starting at 0800 and ending at 2000 (seven measurement sets). During this time, the participants did normal activities, mostly studying, with no intervening exercise. Triplicate TDC medial calf measurements were done while seated on the left leg only at a site located 8 cm proximal to the mid-medial malleolus.

Analyses

Statistical estimates of the differences in TDC values between paired legs and between the initial and final TDC values after 60 minutes of supine lying (goal 1) were determined using the nonparametric Wilcoxon test. Investigation of the possible temporal variations in TDC values measured over the 0800 to 2000 time interval (goal 2) was evaluated via linear regression. In both analyses, a statistical significance was judged based on a p-value < 0.05.

## Results

TDC bilateral and temporal comparisons

Table 1 summarizes bilateral paired-leg TDC values at the medial leg site measured initially and again after 60 minutes of supine lying. TDC values were not different between legs at the initial or final measurements (p>0.600). However, TDC values decreased slightly on both legs (p<0.05), but inter-leg ratios did not change during the 60 minutes of supine lying. The percentage decrease in TDC over the 60-minute interval was 4.6% ± 5.2% for the left leg and 2.1% ± 1.9% for the right leg. The inter-leg TDC ratio (left leg/right leg) was insignificantly changed over the 60-minute lying interval being 0.996 ± 0.035 initially and 0.971 ± 0.067 finally. Averaging initial and final inter-leg ratios yields a composite left/right inter-leg ratio of 0.983 ± 0.054. Adding 3SD to this ratio yields a threshold ratio of about 1.15. If the inter-leg ratio is calculated as right/left, then the average ratio is 1.020 ± 0.060, and the 3SD threshold is 1.20. This threshold may be interpreted as an inter-leg TDC ratio that, if exceeded, may suggest the presence of lower extremity lymphedema. However, given the small number of subjects who are included, such a threshold is only an initial estimate.

**Table 1:** Paired-Leg TDC Values at the Start and After 60 Minutes of Supine Lying Data are mean ± SD with p-values determined from Wilcoxon signed-rank tests. There was no significant difference in TDC values between legs at the initial or final measurements made after one hour of supine lying. TDC values decreased slightly from the initial to the final measurements for both legs. The asterisk (*) denotes a significant difference between the final and the initial measurements (p<0.05). The inter-leg ratio did not significantly change from the initial to the final measurements.

	TDC values						
	Initial		Final		Left/right TDC ratios		
	Left legs	Right legs	Left legs	Right legs	Initial	Final	
TDC	27.7 ± 2.2	27.8 ± 2.7	26.4 ± 2.6*	27.2 ± 2.0*	0.996 ± 0.035	0.971 ± 0.067	
p-value	0.759		0.645		0.169		

TDC variation over time

**Figure 2:**
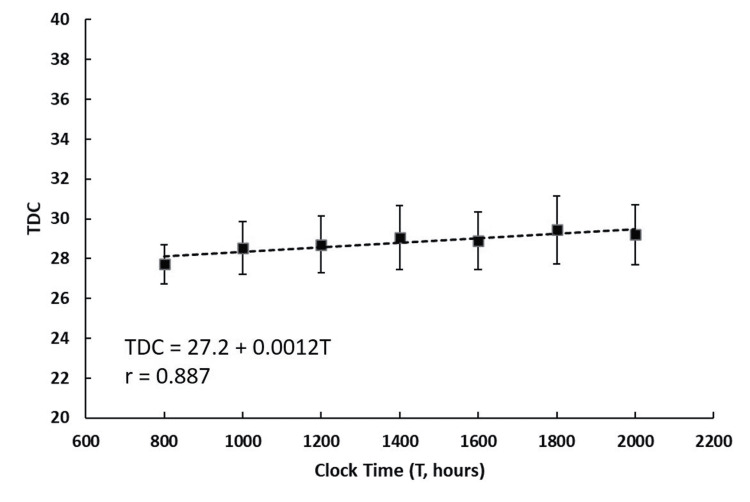
TDC Temporal Variations Data points are the mean value of TDC ± standard error of the mean (SEM) for 10 female subjects self-measured on their medial calf, 8 cm proximal to the medial malleolus at two-hour contiguous intervals. A slight rise in TDC value is indicated by the linear regression line (dashed) with the equation shown in the figure.

Figure 2 shows the TDC variation over the 12-hour measurement interval. Based on regression analysis, there was a slight, but statistically significant, increase in TDC over this interval (r = 0.887, p = 0.008). The TDC values at 0800 and 2000 hours were 27.7 ± 3.1 and 29.2 ± 4.7, respectively, amounting to an increase in TDC of 5.4% over the 12-hour interval.

## Discussion

The focus of the present study was to provide quantitative information on normal lower extremity TDC values including their inter-leg difference and their temporal variation. The main purpose was to provide some initial guidance as to likely variations among healthy women as a reference for the use of TDC measurements in women with unilateral or bilateral edema or lymphedema. A compelling reason for this investigation was that when TDC measurements are used to assess the extent of localized lymphedema and to track and characterize treatment effects, such information would be valuable. The approach adopted was to use two groups of women in which different aspects could be systematically evaluated.

In this initial undertaking, the inclusion of only women is consistent with the fact that lower extremity lymphedema is more prevalent in women than in men with its occurrence reported in up to 70% of women who have been treated for gynecological malignancies [30]. In a retrospective study of 440 lymphedema cases, it was reported that overall, 71% were women [31], and in another study of 76 lower extremity lymphedema cases assessed via magnetic resonance angiography, 83% were women [32]. However, the female/male proportion was reported to be more similar when cases were defined as leg swelling associated with venous hypertension [33]. Other studies have found no clear differentiation between the leg fluid distribution of lower extremity lymphedema versus lower extremity edema associated with venous insufficiency [34]. In the present study, it was felt that for this initial work, the study of a single gender would be more efficient and would help minimize gender-specific covariates.

As part of goal 1, inter-leg TDC differences and their change during one hour of supine lying were determined. This information has potential clinical relevance since lower extremity lymphedema treatments and evaluations occur over this time frame. The findings indicate only minor changes in TDC over this interval with no significant change in inter-leg ratios. As part of goal 2, day-to-night TDC variations were assessed via multiple sequential measurements. This information bears on the potential impact of time-of-day influence on TDC values. The results indicate a small decrease of about 5% over the entire 12-hour measurement interval but that the time-of-day variation is small during daylight hours.

Although the number of subjects evaluated was not large, the findings do provide insight into the range of expected temporal variations for TDC as measured on the legs of healthy women and normal values of inter-leg TDC ratios. To the extent that the inter-leg TDC ratio herein found reflects that which would be present in a larger non-edematous population, the ratio may provide a useful estimate of the presence of unilateral lymphedema as has been done for upper extremity unilateral lymphedema [12-14]. In those studies, the presence of unilateral lymphedema was judged to be likely when the TDC ratio (at-risk arm/contralateral arm) exceeded the mean value of the healthy inter-arm ratio plus 2.5 or 3.0 SD. Applied to the present data, using a 3SD threshold, it suggests the presence of lower extremity lymphedema if a patient’s inter-leg ratio exceeds 1.20. This value is similar to the value of 1.26 previously determined for upper extremity lymphedema [35]. It remains to evaluate the utility of this threshold ratio in patients with unilateral lower extremity edema or lymphedema.

The principal limitation of this study’s findings is the relatively small number of subjects included and evaluated. A secondary limitation is that because the studied group included only women, the findings for men may differ in any aspect. In future studies, the inclusion of a greater number of subjects of both sexes would seem useful to evaluate the generalizability of the present findings.

## Conclusions

The findings indicate that TDC values, as an assessment parameter of lower extremity localized skin-to-fat tissue water, minimally depend on potential confounding factors such as the duration of supine lying and the time of day when the measurements are made. Based on a small sample size, an inter-leg TDC ratio exceeding a value of 1.2 is suggested as a potential threshold for judging unilateral lower extremity edema or lymphedema. Verifying this threshold in patients with lower extremity edema or lymphedema is suggested.
